# Using the WHO Essential Medicines List to Assess the Appropriateness of Insurance Coverage Decisions: A Case Study of the Croatian National Medicine Reimbursement List

**DOI:** 10.1371/journal.pone.0111474

**Published:** 2014-10-22

**Authors:** Antonia Jeličić Kadić, Maja Žanić, Nataša Škaričić, Ana Marušić

**Affiliations:** 1 Department of Anatomy, University of Split School of Medicine, Split, Croatia; 2 Department of Gynaecology and Obstetrics, University of Split Hospital Center, Split, Croatia; 3 School of Humanities and Social Sciences, University of Zagreb, Zagreb, Croatia; 4 Department of Research in Biomedicine and Health, University of Split School of Medicine, Split, Croatia; Canadian Agency for Drugs and Technologies in Health, Canada

## Abstract

**Purpose:**

To investigate the use of the WHO EML as a tool with which to evaluate the evidence base for the medicines on the national insurance coverage list of the Croatian Institute of Health Insurance (CIHI).

**Methods:**

Medicines from 9 ATC categories with highest expenditures from 2012 CIHI Basic List (n = 509) were compared with 2011 WHO EML for adults (n = 359). For medicines with specific indication listed only in CIHI Basic List we assessed whether there was evidence in Cochrane Database of Systematic Reviews questioning their efficacy and safety.

**Results:**

The two lists shared 188 medicines (52.4% of WHO EML and 32.0% of CIHI list). CIHI Basic List had 254 medicines and 33 combinations of these medicines which were not on the WHO EML, plus 14 medicines rejected and 20 deleted from WHO EML by its Evaluation Committee. For deleted medicines, we could obtain data that showed 2,965,378 prescriptions issued to 617,684 insured patients, and the cost of approximately € 41.2 million for 2012 and the first half of 2013, when the CIHI Basic List was in effect. For CIHI List-only medicines with a specific indication (n = 164 or 57.1% of the analyzed set), fewer benefits or more serious side-effects than other medicines were found for 17 (10.4%) and not enough evidence for recommendations for specific indication for 21 (12.8%) medicines in Cochrane systematic reviews.

**Conclusions:**

National health care policy should use high-quality evidence in deciding on adding new medicines and reassessing those already present on national medicines lists, in order to rationalize expenditures and ensure wider and better access to medicines. The WHO EML and recommendations from its Evaluation Committee may be useful tools in this quality assurance process.

## Introduction

Essential medicines are those that satisfy the priority health care needs of a population [1]. They are selected with regard to their public health relevance, evidence on efficacy and safety, and comparative cost-effectiveness. The first global essential medicines list (EML) was assembled in 1977 by the World Health Organization (WHO), which is revised every other year [2]. Medicines are identified through an evidence-based process in which quality, safety, efficacy and cost-effectiveness are key selection criteria [3]. Although the Model List was not designed as a global standard, it has contributed to global acceptance of the concept of essential medicines and can be used by countries as a guide for the development of their own national essential medicines list [2], [4]–[6]. National essential medicines may be helpful in informing decisions in insurance coverage, as they help effective allocation of often limited financial resources, which is important because medicines spending in many countries amounts to about 17% of total health spending or 1.5% of gross domestic product [7].

The WHO's record of national medicines list has 117 countries [8], including most of the countries in the Central, East and South Europe (Bulgaria, Croatia, Macedonia, Montenegro, Poland, Moldova, Serbia, Slovakia, Slovenia, and Ukraine). These countries underwent fundamental economic and political changes after the breakdown of state socialism, which also affected the policy, ownership and financing of health care, mostly in primary health care [9], [10]. In Croatia, which has universal health care coverage, there is no separate national medicines list, but only the insurance coverage list composed by the Croatian Institute for Health Insurance (CIHI). This list (i.e., its 2010 version) is filed as the Croatian national list in the WHO collection of National Medicines List/Formulary/Standard Treatment Guidelines [8].

CIHI is the public insurance fund that dominates both the mandatory and supplementary health insurance in the Croatian health care system, where the impact of the private health insurance on the market is minimal at the moment [11]. CIHI was established in 1993, based on the Health Care Law, and is responsible for the implementation of health policies and financing and control of health services, including all medicines prescribed by primary health care doctors and those dispensed in pharmacies [11]. CIHI compiles two lists of medicines – the Basic List, where medicines are fully reimbursed by the CIHI, and the Supplementary List, for which the patients have to pay a fraction of the cost. Once a drug is registered in Croatia, its manufacturer may apply to have the drug placed on the Basic or Supplementary List for reimbursement coverage by the CIHI. The drug is reviewed by the Committee for Medicines, appointed by the CIHI Governing Board [12]. The Committee includes representatives from the CIHI, The Ministry of Health, the Croatian National Institute of Public Health, as well as clinical pharmacologists and clinicians.

The aim of our study was to assess the use of the WHO EML as a tool with which to evaluate the evidence base for those medicines on the Croatian national insurance coverage list. We also compared the recommendations on medicines rejected or deleted from the WHO EML by the WHO Expert Committee on the Selection and Use of Essential Medicines. To evaluate the rationale for including and maintaining medicines on CIHI Basic List which were not present in the WHO EML we checked whether there was evidence questioning their efficacy and safety in systematic reviews produced by Cochrane Collaboration, which are considered the best quality guidance for clinical practice [13].

## Methods

### Data Collection

The sources of data included the 17^th^ edition of the WHO Model List of Essential Medicines (WHO EML) for adults from 2011 (prepared by the WHO Expert Committee in March 2009 and revised in January 2010) and the CIHI Basic List of medicines which was in effect in 2012. Both lists are publicly available online on official websites of the WHO [1] or CIHI [14], respectively. The terminology used in both lists is from the International Nonproprietary Names, INN (generic names) for medicines [15].

In the WHO Model List, drugs and medical products are divided into 29 therapeutic classes. Some medicines in the WHO EML with a clinically-equivalent pharmacological class are listed with a square box symbol (□) preceding the drug name [1]: “The listed medicine should be the example of the class for which there is the best evidence for effectiveness and safety. In some cases, this may be the first medicine that is licensed for marketing; in other instances, subsequently licensed compounds may be safer or more effective. Where there is no difference in terms of efficacy and safety data, the listed medicine should be the one that is generally available at the lowest price, based on international drug price information sources.” For the purpose of this study, if a medicine was listed on the WHO EML with a square box, all medicines in the same pharmacological class were also considered to be listed on the WHO EML.

The CIHI Basic List is sorted by the Anatomical Therapeutic Chemical (ATC) classification [16] into 14 groups according to the organ or system on which they act and their therapeutic, pharmacological and chemical properties. We identified 9 ATC classes of drugs which are most commonly prescribed and with the highest annual CIHI reimbursement according to the 2011 Chartbook of the Agency for Medicines and Medicinal Products [17]: 1) A – Alimentary tract and metabolism, 2) B – Blood and blood forming organs, 3) C – Cardiovascular system, 4) G – Genito-urinary system and sex hormones, 5) J – Antiinfectives for systemic use, 6) L – Antineoplastic and immunomodulating agents, 7) M – Musculo-skeletal system, 8) N – Nervous system, and 9) R – Respiratory system. ATC groups D – Dermatologicals, H – Systemic hormonal preparations, excluding sex hormones and insulins, P – Antiparasitic products, insecticides and repellents, S – Sensory organs, and V – Various were not included in the analysis.

The 9 ATC classes selected from the CIHI list were compared with all medicines on the WHO EML list for adults because of the difference in the sorting systems between the two lists, which did not allow reliable matching. Because the CIHI Basic List does not separately identify medicines for the use in children, the WHO EML for children was not included in this study.

### Comparison between the WHO Model List and CIHI Basic List

Medicines on the CIHI Basic List were compared with the WHO EML to identify: i) medicines on both lists (in the same or different dose, formulation and/or indication), ii) medicines only on the WHO EML, and iii) medicines only on the CIHI Basic List. Where a difference was identified, we searched the WHO EML Technical Report Series [18], for reference to the EML decision (deletion or rejection) for the indication of the medicine included in the CIHI list. Where possible, a note was made of the recommendation from the meeting of the Expert Committee on the Selection and Use of Essential Medicines.

The data on the expenditures for medicines from the CIHI Basic List are not publicly available for individual medicines. For the subset of medicines on the CIHI Basic List that were deleted by the WHO Expert Committee, we were able to obtain the data from CIHI on the number of prescribed medicines by primary health care physicians and dispensed by pharmacies and their cost to CIHI in 2012 and the first 6 months of 2013, when the CIHI Basic List evaluated in this study was in effect (the new list was introduced in August 2013). We were not able to obtain data for the medicines rejected by the Expert Committee or for individual medicines on the total CIHI Basic List.

### Evidence Base for Medicines Present Only on CIHI List

For medicines included only in the CIHI Basic List and with specified indications, we checked for evidence to support decisions regarding efficacy and safety. Our intention was not to find all available evidence for each specific medication but to see whether there were medicines for which contraindications outweighed benefits for specified indications. To make this exercise more stringent, we searched only the Cochrane Database of Systematic Reviews (CDSR) because Cochrane reviews are generally considered to provide highest quality evidence to inform clinical practice [13]. In addition, the “Ordinance on establishing the criteria for inclusion of medicines in the basic and the supplementary reimbursement list of the Croatian Institute for Health Insurance” requires that the application for the inclusion of a medicine on the list has to present evidence based on the search of at least the Cochrane Library, including CDSR, and the PubMed databases [12].

The search strategy included the generic name of the medicine and medical indication, including the variations in both sets of terms. From the retrieved list of articles, we analyzed the full text of the most recent update of any given systematic review, with the time limit set up to 2012, when the CIHI Basic List was published.

## Results

WHO EML (n = 359) was compared with 509 (79.4%) medicines from 9 ATC classes from the total of 641 medicines on the CIHI Basic List. There were 188 medicines that were found on both lists (52.4% of the WHO EML and 36.9% of the CIHI list). Among these, 65 (34.6%) medicines in the CIHI Basic List had the same dose formulation and indication as in the WHO EML, 46 (24.5%) medicines had a different dose, 16 (8.5%) had a different formulation and 7 (3.7%) had a different indication than the medicines in the WHO EML (**Table 1**). Further, 54 (28.7%) medicines in the CIHI Basic List had differences in more than one characteristic (dose, formulation and/or indication) than their counterparts in the WHO EML (**Table 1**).

**Table 1:** Comparison of the Basic List of medicines from the Croatian Institute for Health Insurance (CIHI) with the WHO Essential Medicines List (EML).

Finding	CV	CNS	GI	ONCOL	INF	BLOOD	RESP	MS	UG	Total
No difference	2	10	3	15	20	10	1	4	0	65
Different dose	5	4	4	11	8	10	2	0	2	46
Different formulation	0	2	0	3	9	0	1	0	1	16
Different indication	1	0	0	0	3	1	1	0	1	7
Different dose & formulation	7	8	4	3	8	2	1	4	2	39
Different dose & indication	1	1	3	0	3	0	0	0	0	8
Different formulation & indication	0	0	0	0	2	0	0	0	0	2
Different dose, formulation & indication	1	1	1	0	2	0	0	0	0	5
Only on EML (and in combinations)*	3 (0)	3 (0)	1 (0)	4 (0)	11 (8)	3 (1)	1 (0)	1 (0)	5 (1)	32 (10)
Only on CIHI list (and in combinations)*	26 (14)	40 (3)	31 (2)	72 (0)	15 (5)	27 (6)	8 (3)	14 (0)	21 (0)	254 (33)
CIHI List, deleted from EML	3	2	2	3	2	2	2	2	2	20
CIHI List, rejected from EML	0	11	0	0	2	0	0	0	1	14

Disease groups: CV – Cardiovascular, CNS – Central nervous system, GI – Gastrointestinal, ONCOL – Oncological and immunomodulatory, INF – Systemic infection, B – Blood/hematopoietic, RESP – Respiratory, MS – Musculoskeletal, UG – Genitourinary.

*The number in brackets represents the combinations of only CIHI or EML medicines, which were also included in the lists.

WHO EML had 32 individual and 10 combinations of the medicines which were not listed on the CIHI Basic List (**Table 1**). The differences were mostly among infectious diseases medicines (**Table 1**), primarily those for the treatment of AIDS (Antiretroviral medicines, n = 7) and tuberculosis (Antituberculosis medicines, n = 11).

The CIHI Basic List had 287 medicines that were not on the WHO EML: 254 individual medicines plus 33 combinations of these medicines. In addition to these medicines, the CIHI Basic List also had 20 medicines that were deleted and 14 medicines that were rejected from the WHO EML, bringing the total number of extra medicines on CIHI Basic List in comparison to WHO EML to 321.

The medicines only on the CIHI Basic List and not on the WHO EML (n = 287) were mostly from the following classes: Antineoplastic and immunomodulating agents (n = 75), mostly non-classified cytostatics (n = 26) and immunosuppressive medicines (n = 13); Nervous system group (n = 56), mostly narcotic medicines (n = 10) and antipsychotic medicines (n = 9); and Cardiovascular system group (n = 43), mostly ACE inhibitors (n = 14).

The CIHI Basic List contained additional 20 medicines (63 separate preparations by 4 pharmaceutical companies) that were deleted from WHO EML (**Table 1**), mostly because of the lack of evidence of efficacy for the specified indication in comparison to existing medicines on the WHO EML (**Table 2**). For this subset of medicines regarding their consumption and cost to the CIHI in 2012 and the first half of 2013, when the Basic List was officially in use, there were 2,965,378 prescriptions for these medicines issued to 617,684 insured patients, amounting to 308,918,281 Croatian Kunas (approximately € 41.2 million).

**Table 2:** Medicines on the Croatian Institute for Health Insurance (CIHI) Basic List that were deleted from the WHO Essential Medicines List (EML).

Medicine/Disease Group	Technical Report Series (TRS), year	Explanation – reason for deletion
albumin/B	TRS895, 2000	The review by the Cochrane Collaboration suggest the likelihood of previously unrecognized hazards and a lack of evidence of better efficacy of albumin compared with alternatives.
aminophyllline/RESP	TRS933, 2005	The Committee recommended that aminophylline and theophylline be deleted from the Model List because of the availability of safer and more effective alternatives on the Model List.
atropine as spasmolytic for gastrointestinal diseases/GI	TRS933, 2005	The Committee therefore recommended that atropine (as an antispasmodic) together with the whole section on antispasmodic medicines be deleted from the Model List because of lack of evidence of efficacy and safety.
busulfan/ONCOL	TRS722 1985	Busulfan and chlorambucil are deleted.
calcium carbonate/GI	TRS770, 1988	Calcium carbonate is deleted since it causes greater gastric secretion and acid rebound than other listed antacids. (on CIHI list recommended for hyperphosphatemia, A12AA04 131).
chlormethine/ONCOL	TRS641, 1979	It was deleted from the main list of antineoplastic and immunosuppressive drugs, since it offers no clear advantage over the other drugs listed.
chlortalidone/CV	TRS770, 1988	Chlortalidone is deleted since the differences between chlortalidone and thiazide diuretics are of minor therapeutic significance.
cisplatin/ONCOL	TRS958 2009	The Committee therefore recommended that carboplatin replace cisplatin on the Complementary Model List (with a square box) for the treatment of advanced ovarian cancer.
clonazepam/CNL	TRS933, 2005	The Expert Committee recommended that clonazepam be deleted because of the lack of evidence of better efficacy or safety when compared with valproate.
colchicine/MS	TRS933, 2005	The Committee recommended that colchicine be deleted from the Model List because of its unfavourable benefit–risk ratio when compared with non-steroidal anti-inflammatory drugs (NSAIDs) for most people with gout.
diazoxide injection/CV	TRS641, 1979	Diazoxide injection: was deleted from the main list of antihypertensive drugs, since it is covered by note after sodium nitroprusside.
doxazosin/CV	TRS895, 2000	Prazosin tablet, 500 f.1g and 1 mg, replaces doxazosin in the complementary list as the representative of the α-adrenoreceptor antagonist class of drugs since it is now less expensive than doxazosin (recommended on CIHI list for hypertension and benign prostate hypertrophy).
ergometrine tablet/UG	TRS933, 2005	There was no robust clinical evidence to establish the effectiveness and safety of ergometrine used alone for active management of labour. There was no clinical trial evidence to support the efficacy and safety of ergometrine used alone or in combination with oxytocin for the treatment of postpartum haemorrhage. The Committee saw no indication for ergometrine tablets (Injections are still on the EML).
estradiol/UG	TRS965 2011	The Committee noted that long-term hormone replacement treatment of menopause is no longer considered appropriate, notwithstanding individuals' possible need for treatment of symptoms.
fibrinogen/B	TRS685, 1983	Fibrinogen and plasma protein injectable solution are deleted from the complementary list (no further information on this deletion is available).
indometacin/MS	TRS867, 1997	Indometacin is deleted from this section since there are many non-steroidal anti-inflammatory drugs with a similar action.
ketoconazole/INF	TRS895 2000	Fluconazole replaces ketoconazole as the prototype drug since it is more cost-effective and is associated with fewer adverse effects.
pethidine/CNL	TRS920, 2003	The Committee noted that pethidine was listed on 19 out of 25 national essential medicine lists; that pethidine was considered inferior to morphine due to its toxicity on the central nervous system; and that it is generally more expensive than morphine. The Committee concluded that there was insufficient justification to keep pethidine on the Model List and recommended that it be deleted. The Committee stressed that all national programmes should ensure that sufficient quantities of morphine are always available for those who need it.
protionamide/INF	TRS770, 1988	Ethionamide and protionamide have been deleted from the complementary list on the grounds that they are rarely required as replacements for clofazimine, which is a less toxic drug.
theophylline/RESP	TRS933, 2005	See aminophylline.

Disease groups: CV – Cardiovascular, CNS – Central nervous system, GI – Gastrointestinal, ONCOL – Oncological and immunomodulatory, INF – Systemic infection, B – Blood/hematopoietic, RESP – Respiratory, MS – Musculoskeletal, UG – Genitourinary.

The CIHI Basic List contained another 14 medicines (150 separate preparations by 10 pharmaceutical companies) that were rejected by the WHO EML Committee; mostly in the central nervous system diseases group (n = 9; **Table 1**). The reasons for rejection were also mostly the lack of evidence for efficacy (**Table 3**).

**Table 3:** Medicines on the Croatian Institute for Health Insurance (CIHI) Basic List that were rejected by the WHO Essential Medicines List (EML).

Medicine/Disease group	Technical Report Series (TRS), year	Explanation – reason for rejection
amantadine/CNL	TRS958, 2009	The Committee recommended not including any of the antivirals on the Model List at the present time. However the Committee endorsed the proposal for an emergency meeting mechanism to consider one or more of the antivirals, including for paediatric use, should a pandemic occur.
clozapine/CNL	TRS958, 2009	The applications did not provide sufficient information regarding the comparative effectiveness and safety of the proposed medicines (clozapine, olanzipine, risperidone, quetiapine, aripiprazole and ziprasidone).
ziprasidone/CNL	TRS958, 2010	
darunavir/INF	TRS965, 2011	Given the relatively limited evidence of efficacy, safety, and cost– effectiveness in both adults and children in a diversity of settings, that the optimal use of darunavir is still being defined, and uncertainty regarding the best combinations of medicines for third-line regimens, the Committee recommended that darunavir should not be added to the Complementary List. Further development of darunavir is clearly required, including fixed-dose combination products of darunavir/ritonavir especially for children.
escitalopram*/CNL	TRS958, 2009	Overall the Committee decided that the evidence provided in the application did not support the public health need or comparative effectiveness, safety and cost-effectiveness for the addition of escitalopram, paroxetine or sertraline to the Model List at this time.
lamotrigine/CNL	TRS958 2009	The Committee did not recommend the inclusion of lamotrigine on the Model List based on the lack of evidence of its superior efficacy and safety and cost-effectiveness with respect to comparators, and the availability of suitable alternative first-line antiepileptics which are already on the Model List. The Committee recommended a review of second-line antiepileptics for a future meeting, including a review of topiramate, lamotrigine and gabapentin as a second-line therapy for children and adults.
levonorgestrel-releasing IUD/UG	TRS933 2005	The Committee recommended rejection of the application for inclusion of the levonorgestrel-releasing IUD for contraception because of the lack of evidence for better efficacy, its higher discontinuation rate and because it is more expensive than the copper IUD already in the Model List.
paroxetine/CNL	TRS958 2009	The Committee decided that the evidence provided was not sufficient to recommend the addition of paroxetine and sertraline or addition of a square box to fluoxetine.
pentazocine/CNL	TRS825 1992, TRS850 1995, TRS867 1997	The Committee has rejected a request to add pentazocin to the list, since it would have been endorsing to use an inferior analgesic for victims of large scale emergencies or disasters because of regulatory requirements. Rather, the Committee strongly urged that administrative and regulatory requirements be modified to permit the use of essential drug morphine in emergency health care.
quetiapine*/CNL	TRS958 2009	The application did not provide sufficient information regarding the comparative effectiveness and safety of the proposed medicines.
raltegravir/INF	TRS 965 2011	Raltegravir was rejected due to the comparatively limited efficacy, safety, and cost–effectiveness in both adults and children in a diversity of settings and because the optimal use of raltegravir is still being defined, as well as the best combinations of medicines for third-line regimens.
risperidone/CNL	TRS882 1998, TRS958 2009	The application did not provide sufficient information regarding the comparative effectiveness and safety of the proposed medicines.
sertraline*/CNL	TRS958 2009	See paroxetine.
sumatriptan/CNL	TRS946 2007, TRS958 2009	Sumatriptan 50 mg tablet – rejected on the grounds that the comparative efficacy, safety and cost-effectiveness of sumatriptan versus other triptans and aspirin were not established.

Disease groups: CV – Cardiovascular, CNS – Central nervous system, GI – Gastrointestinal, ONCOL – Oncological and immunomodulatory, INF – Systemic infection, B – Blood/hematopoietic, RESP – Respiratory, MS – Musculoskeletal, UG – Genitourinary.

*Newly added to CIHI list.

Out of 287 medicines that were solely on CIHI List (not including WHO EML deleted or rejected medicines), specific indication was available for 164 (57.1%) medicines (**Table 4**). For this subset of CIHI List medicine, a Cochrane systematic review was not available for 50 medicines (30.5%), and for 33 medicines (20.1%) there was a Cochrane systematic review available but it did not include the specified indication. For 41 (25.0%) medicines there was evidence for the same or more benefits than comparators. Fewer benefits or more serious side-effects than other medicines were found for 17 (10.4%) medicines, and there was not enough evidence for the recommendations for specific indications for 21 (12.8%) medicines. We also found that the recommendations for 2 medicines and their indications were removed due to obsolete Cochrane systematic reviews (**Table 4**). Detailed information on the evidence from Cochrane Systematic reviews is available in **Table S1**.

**Table 4:** Evidence from Cochrane systematic reviews on medicines on the Croatian Institute for Health Insurance (CIHI) Basic List for medicines that were not on WHO Essential Medicines List (EML) (n = 287).

Finding	CV	CNS	GI	ONCOL	INF	BLOOD	RESP	MS	UG	Total
No specific indication on CIHI list	25	26	8	20	11	14	5	6	8	123
No CSR available for specific medicine	9	2	9	17	3	4	1	4	1	50
No CSR for indication on CIHI list	3	3	5	7	3	7	1	2	2	33
Same or more benefits as other medicines	0	10	1	18	1	4	3	1	3	41
Ineffective or fewer benefits than other medicines	3	0	2	4	2	2	0	0	3	16
Same or more benefits as other medicines but serious side effects	0	0	0	1	0	0	0	0	0	1
Not enough evidence for conclusion about specific indication in CSR	0	2	8	5	0	2	1	1	2	21
CSR not updated*	0	0	0	0	0	0	0	0	2	2

Disease groups: CV – Cardiovascular, CNS – Central nervous system, GI – Gastrointestinal, ONCOL – Oncological and immunomodulatory, INF – Systemic infection, B – Blood/hematopoietic, RESP – Respiratory, MS – Musculoskeletal, UG – Genitourinary.

*CSR not updated – the review has been withdrawn until its authors update it.

## Discussion

Our study showed that the WHO EML, along with the recommendations by its Selection Committee, can be a useful tool in evaluating national medicines lists by indicating which medicines are considered to have adequate supporting evidence for decision regarding their inclusion on the list. The Croatian national medicines list, i.e. its Basic List for mandatory insurance reimbursement by the CIHI, included 34 medicines among most commonly prescribed ATC classes (7% of 509 medicines) which were deleted or rejected from the WHO EML. This finding, despite prescribed reliance on evidence in decisions about the selection of medicines for the Croatian national list [12], calls into question the effectiveness of the listed drugs: is effectiveness indeed based on sound evidence and international standards? Moreover, findings from high-quality systematic reviews showed that almost a quarter of the medicines with specific indications which were unique to the CIHI Basic List contained 10.4% medicines where the risks or side-effects outweighed the benefits when examined alongside comparator medicines or for which there was insufficient evidence (12.8% medicines) for drug recommendations for specific indications. For the subset of medicines deleted from the WHO EML but present on the CIHI Basic List, the Croatian national health insurance spent about €40 million during the 1.5 years of its use. In the total pharmaceutical expenditure in Croatia, estimated at about 676 million Euros in 2012 [19], this sum is considerable and may be perceived as an investment in medicines that may be offering limited overall benefit and potential harm, as well as a missed opportunity for investment in drugs that may offer more benefit for conditions or areas not adequately covered by the CIHI Basic List.

The results of this study should be considered with caution as there are several limitations. Primarily, the cross-sectional as opposed to a longitudinal design did not allow for the evaluation of possible trends in the changes to the national mandatory reimbursement list. We could not obtain the full dataset for the expenditures for all individual medicines on the CIHI Basic List to judge the relationship between their cost and evidence for inclusion in the list. Finally, we searched only the Cochrane Database of Systematic Reviews for supporting evidence about additional medicines included only in the CIHI Basic List. Our conclusions on the evidence of efficacy and safety for these medicines are limited because we used Cochrane systematic reviews as a surrogate marker of effectiveness and we did not evaluate other sources of evidence, such as potential newer randomized controlled trials or clinical practice guidelines, which may better reflect health care practices. Subsequently, this restricted search resulted in the availability of systematic reviews for only half of the CIHI list-only medicines with specific indications. However, the main aim of our search was to identify whether there was evidence from Cochrane systematic reviews, considered to provide highest quality evidence to inform clinical practice [13], to question the inclusion of a medicine on the national mandatory basic medicine list. The fact that we identified Cochrane systematic reviews that showed fewer benefits, more serious adverse effects or not enough evidence for a specified indication for more than 20% of the medicines included in the search, indicates that the decision on placing or maintaining medicines on the CIHI Basic List is not fully based on evidence, despite legal requirements [12] and sufficient decision-making expertise of the CIHI Committee. The 13-member Committee includes clinical pharmacologists, clinicians, public health experts and representatives from the Ministry of Health and the CIHI, and evaluates evidence from different sources, including individual studies and non-Cochrane reviews [12].

Although the decisions on the inclusion of medicines on the CIHI lists are made at regular monthly meetings of the CIHI Committee and are published on the CIHI website, the evidence on which the decisions are based or complete applications from drug manufacturers is not provided. The finding that the CIHI Basic List contained 34 medicines (7% of 509 medicines in 9 analyzed ATC categories) which were deleted or rejected from the WHO EML may be explained by the focus of the CIHI Committee on approving new medicines rather than systematically monitoring medicines already on the list. Judging from the recommendations on applications from monthly Committee meetings (available at http://www.hzzo.hr/zdravstveni-sustav-rh/pravilnik-o-mjerilima-za-stavljanje-lijekova-na-osnovnu-i-dopunsku-listu), this seems to be the case. It was not possible to determine whether the 34 medicines from this study were historically present on the CIHI Basic List since the online archive of the basic and supplementary lists provides data up to 2012, and the 2011 basic list is included in the WHO collection of national medicines lists [8]; we did not have access to the printed issues of the lists. The Ordinance [12] specifies that medicines can be deleted from the lists based on the recommendation of the Committee not only when the applicant requests such removal or when the medicinal production is discontinued or the medicine is not available on the market for more than 6 months, but also based on expert opinion that there is no justification for further use or if the Ministry of Health or the Agency for Medicines and Medical Devices establish harmful effects of a medicine. Thus, it seems that the decision-making aspect concerning drugs on the Croatian national medicines lists is not fully functional. If the regulatory bodies involved in the process – the Ministry of Health, the CIHI and the Agency for Medicines and Medical Devices – consulted Technical Reports of the WHO EML Committee they could have identified the cases for possible re-evaluation of medicines in order to increase rationality and cost-savings of the national medicines list. After our study was completed, the CIHI published the new Basic List, implemented from August 2013, which still contained medicines deleted or rejected from the WHO EML.

CIHI Basic List for the 9 ATC categories in this study matched 52% of the whole WHO EML. There were also 12% of medicines from the WHO EML that were not on the CIHI Basic List for 9 ATC categories. This means that 36% of the WHO EML was not related to most commonly prescribed medicines in Croatia, including the ATC categories Dermatologicals, Antiparasitic products, insecticides and repellents, and Sensory organs. Medicines that were on the WHO EML but not CIHI Basic List were predominantly those for infectious diseases – mostly HIV and tuberculosis. This finding reflects low prevalence of HIV (<0.1% of the adult population [20]) and relatively low prevalence of tuberculosis (22/100,000 population [21]) in Croatia. It also reflects separate legal regulation of health care for specific diseases or population groups. According to the Law on Mandatory Health Care of the Republic of Croatia [22], Law on the Protection of Public from Infectious Diseases [23] and Law on Mandatory Health Care [22], full health care coverage is provided for AIDS and other infectious diseases of public health importance.

The finding that the Croatian basic national medicines list included a total of 321 medicines (63% of the total analyzed medicines from the 9 most commonly prescribed ATC classes) that were not present in the WHO EML is not surprising for a high-income country, as Croatia is classified by the World Bank [24]. WHO EML cannot serve as a strict model for national medicines lists and differences are expected and justifiable from the local geographical or socio-economical point of view [2]. For Croatia, the burden of non-communicable, chronic diseases, including cancer [25], definitely influences the composition of both the mandatory and supplementary national medicines list. This explains why the greatest fraction of medicines unique to the CIHI Basic List was from the classes of neoplastic, cardiovascular and nervous system diseases. The increase of non-communicable diseases in the developing world [26] is a pertinent public health problem and it can be expected that the WHO EML will also include more medicines for these disease groups.

Cost does not seem to be the driving factor for inclusion in the CIHI Basic List, which guarantees reimbursement for all persons with national health insurance coverage. For example, the CIHI Basic List had 2605 entries from more than 160 different providers for 509 medicines in 9 analyzed ATC groups. We could not make the full assessment of the cost-effectiveness of medicines included in the CIHI Basic List because we could not obtain the full dataset for the spending and expenditures for individual medicines of 9 ATC classes included in the analysis. Pragmatically, the process of pricing is rather complex, so that the price listed in the CIHI Basic List may differ from the actual price paid but the CIHI because of complex rebate and bundling arrangements with the pharmaceutical companies [11]. We could obtained expenditure data only for medicines on the CIHI list which were deleted from the WHO EML, for which Croatia spent about 40 million Euros over the time when the CIHI Basic List from this study was in effect. Just like the WHO EML, CIHI considers comparative effectiveness for medicines on the list [12], so that EML-deleted or rejected medicines should probably not have been included on the Croatian national medicines list. Expenditure for such medicines is a significant problem for the system of health care that is burdened by over-utilization of and over-expenditures for medicines [11] and subjected to constant reforms to achieve financial sustainability and efficiency with limited resources [11].

Evidence for the efficacy may also not be a primary motivator for decisions about the Croatian national medicines list, despite proclaimed principles of evidence-informed decision-making [12]. Our study demonstrated that for at least some of the medicines on the basic national list there was evidence that the harms outweigh the benefits versus their comparators for specific indications. The recommendations of the CIHI Committee should be officially based on 1) importance of the medicine from a public health point of view, 2) therapeutic importance, 3) relative therapeutic value, and 4) ethical aspects [12]. However, there is little transparency in the decision making process for the recommendations of the CIHI Committee about the new or existing medicines on the list. The process is further burdened by possible conflict of interest introduced by the requirement that any application for inclusion on CIHI lists should be accompanied by a paid opinion of a professional expert [12].

In conclusion, our study demonstrated the value of WHO EML and the recommendations of its Expert Committee in evaluating a national medicines list, which is also the national insurance mandatory reimbursement list in Croatia. We identified a number of cases where medicines were included in the national mandatory list against the evidence from an international standard such as the WHO EML. There is also insufficient transparency of the decision-making for inclusion or deletion of medicines from the list. There are examples from both developed and developing country settings for successful implementation of essential medicines principle in practice [2], [27], [28]. In the current situation of growing demands and reducing financial resources for national health care coverage, greater reliance on a well-established and evidence-based national list is essential. Independent, conflict-free, high-quality evidence should be used to support decision-making for medicines reimbursement. This process should be maximally transparent, with decisions publicly available and discussed, and effectively disseminated to all stakeholders.

## Supporting Information

Table S1 Detailed information on available evidence for efficacy of medicines on Croatian Institute for Health Insurance (CIHI) Basic List which are not on WHO Essential Medicines List.
(DOCX)
