# Supplementary material for: Using the WHO Essential Medicines List to Assess the Appropriateness of Insurance Coverage Decisions: A Case Study of the Croatian National Medicine Reimbursement List
Source: PLoS One. 2014 Oct 22;9(10):e111474. doi: 10.1371/journal.pone.0111474 (PMC4206465; doi:10.1371/journal.pone.0111474)
Supplement: Table S1 — Detailed information on available evidence for efficacy of medicines on Croatian Institute for Health Insurance (CIHI) Basic List which are not on WHO Essential Medicines List. (DOCX) [file pone.0111474.s001.docx]

**Supplementary Material:** Jeličić Kadić A, Žanić M, Škaričić N, Marušić A. Using the WHO Essential Medicines List to assess the appropriateness of insurance coverage decisions: A case study of the Croatian national medicine reimbursement list

Corresponding author: [ana.marusic@mefst.hr](mailto:ana.marusic@mefst.hr)

**Table S1.** Detailed information on available evidence for efficacy of medicines on Croatian Institute for Health Insurance (CIHI) Basic List which are not on WHO Essential Medicines List

| **Medicine** | **Source of evidence:** |
| --- | --- |
| **Cardiovascular:** |  |
| sotalol | Arsenault KA et al. Interventions for preventing post-operative atrial fibrillation in patients undergoing heart surgery. Cochrane Database Syst Rev 2013;31;1: CD003611 |
| losartan | Heran BS et al. Blood pressure lowering efficacy of angiotensin receptor blockers for primary hypertension. Cochrane Database Syst Rev 2009;17;8: CD003822  Heran BS et al. Angiotensin receptor blockers for heart failure. Cochrane Database Syst Rev 2012;18;4: CD003040 |
| valsartan | Heran BS et al. Blood pressure lowering efficacy of angiotensin receptor blockers for primary hypertension. Cochrane Database Syst Rev 2009;17;8: CD003822  Heran BS et al. Angiotensin receptor blockers for heart failure. Cochrane Database Syst Rev 2012;18;4: CD003040 |
| **Central nervous system:** |  |
| [vigabatrin](http://www.whocc.no/atc_ddd_index/?code=N03AG04&showdescription=yes) | Hemming K et al. Vigabatrin for refractory partial epilepsy. Cochrane Database Syst Rev 2013;31;1: CD007302 |
| [topiramate](http://www.whocc.no/atc_ddd_index/?code=N03AX11&showdescription=yes) | Jette N et al. Topiramate add-on for drug-resistant partial epilepsy. Cochrane Database Syst Rev 2009;21;1:CD001417 |
| [gabapentin](http://www.whocc.no/atc_ddd_index/?code=N03AX12&showdescription=yes) | Marson GA et al. Gabapentin add-on for drug-resistant partial epilepsy. Cochrane Database Syst Rev 2009:7;8: CD001415 |
| [pregabalin](http://www.whocc.no/atc_ddd_index/?code=N03AX16&showdescription=yes) | Pulman J et al. Pregabalin add-on for drug-resistant partial epilepsy. Cochrane Database Syst Rev 2009;12;6: CD005612 |
| [pramipexole](http://www.whocc.no/atc_ddd_index/?code=N04BC05&showdescription=yes) | Clarke CE et al. Pramipexole for levodopa-induced complications in Parkinson's disease. Cochrane Database Syst Rev 2009;23;1: CD002261 |
| [sertindole](http://www.whocc.no/atc_ddd_index/?code=N05AE03&showdescription=yes) | Lewis R et al. Sertindole for schizophrenia. Cochrane Database Syst Rev 2009;21;1: CD001715 |
| olanzapine | Komossa K et al. Olanzapine versus other atypical antipsychotics for schizophrenia. Cochrane Database Syst Rev 2010;17;3: CD006654 |
| [quetiapine](http://www.whocc.no/atc_ddd_index/?code=N05AH04&showdescription=yes) | Komossa1 K et al. Quetiapine versus other atypical antipsychotics for schizophrenia. Cochrane Database Syst Rev 2010;20;1: CD006625 |
| [amisulpride](http://www.whocc.no/atc_ddd_index/?code=N05AL05&showdescription=yes) | Komossa K et al. Amisulpride versus other atypical antipsychotics for schizophrenia. Cochrane Database Syst Rev 2010;20;1: CD006624 |
| [zolpidem](http://www.whocc.no/atc_ddd_index/?code=N05CF02&showdescription=yes) | Hirst A et al. Benzodiazepines and related drugs for insomnia in palliative care. Cochrane Database Syst Rev 2009;7;8: CD003346 |
| [buprenorphine, combinations](http://www.whocc.no/atc_ddd_index/?code=N07BC51&showdescription=yes) | Minozzi S et al. Maintenance treatments for opiate dependent adolescent. Cochrane Database Syst Rev 2009;15;4: CD007210 |
| [riluzole](http://www.whocc.no/atc_ddd_index/?code=N07XX02&showdescription=yes) | Miller R et al. Riluzole for amyotrophic lateral sclerosis (ALS)/motor neuron disease (MND). Cochrane Database Syst Rev 2012;14;3: CD001447 |
| **Gastrointestinal:** |  |
| [granisetron](http://www.whocc.no/atc_ddd_index/?code=A04AA02&showdescription=yes) | Billio A. Serotonin receptor antagonists for highly emetogenic chemotherapy in adults. Cochrane Database Syst Rev 2010;20;1: CD006272 |
| [tropisetron](http://www.whocc.no/atc_ddd_index/?code=A04AA03&showdescription=yes) | Billio A. Serotonin receptor antagonists for highly emetogenic chemotherapy in adults. Cochrane Database Syst Rev 2010;20;1: CD006272 |
| [palonosetron](http://www.whocc.no/atc_ddd_index/?code=A04AA05&showdescription=yes) | Billio A. Serotonin receptor antagonists for highly emetogenic chemotherapy in adults. Cochrane Database Syst Rev 2010;20;1: CD006272 |
| [ursodeoxycholic acid](http://www.whocc.no/atc_ddd_index/?code=A05AA02&showdescription=yes) | Rudic JS et al. Ursodeoxycholic acid for primary biliary cirrhosis. Cochrane Database Syst Rev 2012;12;12: CD000551 |
| [insulin lispro](http://www.whocc.no/atc_ddd_index/?code=A10AB04&showdescription=yes) (short acting- SA) | Siebenhofer A et al. Short acting insulin analogues versus regular human insulin in patients with diabetes mellitus. Cochrane Database Syst Rev 2009;21;1: CD003287 |
| [insulin aspart](http://www.whocc.no/atc_ddd_index/?code=A10AB05&showdescription=yes) (SA) | Siebenhofer A et al. Short acting insulin analogues versus regular human insulin in patients with diabetes mellitus. Cochrane Database Syst Rev 2009;21;1: CD003287 |
| [insulin glulisine](http://www.whocc.no/atc_ddd_index/?code=A10AB06&showdescription=yes) (SA) | Siebenhofer A et al. Short acting insulin analogues versus regular human insulin in patients with diabetes mellitus. Cochrane Database Syst Rev 2009;21;1: CD003287 |
| [insulin lispro](http://www.whocc.no/atc_ddd_index/?code=A10AD04&showdescription=yes) (long acting-LA) | Siebenhofer A et al. Short acting insulin analogues versus regular human insulin in patients with diabetes mellitus. Cochrane Database Syst Rev 2009;21;1: CD003287 |
| [insulin aspart](http://www.whocc.no/atc_ddd_index/?code=A10AD05&showdescription=yes) (LA) | Siebenhofer A et al. Short acting insulin analogues versus regular human insulin in patients with diabetes mellitus. Cochrane Database Syst Rev 2009;21;1: CD003287 |
| [insulin glargine](http://www.whocc.no/atc_ddd_index/?code=A10AE04&showdescription=yes) (LA) | Siebenhofer A et al. Short acting insulin analogues versus regular human insulin in patients with diabetes mellitus. Cochrane Database Syst Rev 2009;21;1: CD003287 |
| [insulin detemir](http://www.whocc.no/atc_ddd_index/?code=A10AE05&showdescription=yes) (LA) | Siebenhofer A et al. Short acting insulin analogues versus regular human insulin in patients with diabetes mellitus. Cochrane Database Syst Rev 2009;21;1: CD003287 |
| **Oncological**: |  |
| temozolomide | Hart MG et al. Temozolomide for High Grade Glioma. Cochrane Database Syst Rev 2013;30;4: CD007415 |
| [pemetrexed](http://www.whocc.no/atc_ddd_index/?code=L01BA04&showdescription=yes) | Green JA et al. Pemetrexed disodium in combination with cisplatin versus other cytotoxic agents or supportive care for the treatment of malignant pleural mesothelioma. Cochrane Database Syst Rev 2009;21;1: CD005574 |
| [cladribine](http://www.whocc.no/atc_ddd_index/?code=L01BB04&showdescription=yes) | Steurer M et al. Purine Antagonists for Chronic Lymphocytic Leukaemia. Cochrane Database Syst Rev 2009;21;1: CD004270 |
| [fludarabine](http://www.whocc.no/atc_ddd_index/?code=L01BB05&showdescription=yes) | Steurer M et al. Purine Antagonists for Chronic Lymphocytic Leukaemia. Cochrane Database Syst Rev 2009;21;1: CD004270 |
| [rituximab](http://www.whocc.no/atc_ddd_index/?code=L01XC02&showdescription=yes) | Bauer K et al. Rituximab, ofatumumab and other monoclonal anti-CD20 antibodies for chronic lymphocytic leukaemia. Cochrane Database Syst Rev 2012;14;11: CD008079 |
| [trastuzumab](http://www.whocc.no/atc_ddd_index/?code=L01XC03&showdescription=yes) | Moja L et al. Trastuzumab containing regimens for early breast cancer. Cochrane Database Syst Rev 2012;18;4: CD006243 |
| [bevacizumab](http://www.whocc.no/atc_ddd_index/?code=L01XC07&showdescription=yes) | Wagner AD et al. Anti-angiogenic therapies for metastatic colorectal cancer. Cochrane Database Syst Rev  2009;8;7: CD005392 |
| [sunitinib](http://www.whocc.no/atc_ddd_index/?code=L01XE04&showdescription=yes) | Coppin C et al. Targeted therapy for advanced renal cell carcinoma. Cochrane Database Syst Rev 2008;8;8: CD006017 |
| [sorafenib](http://www.whocc.no/atc_ddd_index/?code=L01XE05&showdescription=yes) | Coppin C et al. Targeted therapy for advanced renal cell carcinoma. Cochrane Database Syst Rev 2008;8;8: CD006017 |
| [temsirolimus](http://www.whocc.no/atc_ddd_index/?code=L01XE09&showdescription=yes) | Coppin C et al. Targeted therapy for advanced renal cell carcinoma. Cochrane Database Syst Rev 2008;8;8: CD006017 |
| [topotecan](http://www.whocc.no/atc_ddd_index/?code=L01XX17&showdescription=yes) | Alvarez MP et al. Chemotherapy versus best supportive care for extensive small cell lung cancer. Cochrane Database Syst Rev 2009;7;8: CD001990 |
| [anastrozole](http://www.whocc.no/atc_ddd_index/?code=L02BG03&showdescription=yes) | Gibson L et al. Aromatase inhibitors for treatment of advanced breast cancer in postmenopausal women. Cochrane Database Syst Rev 2009;7;8: CD003370 |
| [letrozole](http://www.whocc.no/atc_ddd_index/?code=L02BG04&showdescription=yes) | Gibson L et al. Aromatase inhibitors for treatment of advanced breast cancer in postmenopausal women. Cochrane Database Syst Rev 2009;7;8: CD003370 |
| [exemestane](http://www.whocc.no/atc_ddd_index/?code=L02BG06&showdescription=yes) | Gibson L et al. Aromatase inhibitors for treatment of advanced breast cancer in postmenopausal women. Cochrane Database Syst Rev 2009;7;8: CD003370 |
| [interferon alfa-2b](http://www.whocc.no/atc_ddd_index/?code=L03AB05&showdescription=yes) | Myers RP et al. Interferon for acute hepatitis C. Cochrane Database Syst Rev 20019;21;1: CD000369 |
| [interferon beta-1a](http://www.whocc.no/atc_ddd_index/?code=L03AB07&showdescription=yes) | La Mantia L et al. Interferon beta for secondary progressive multiple sclerosis. Cochrane Database Syst Rev 2012;18;1: CD005181 |
| [glatiramer acetate](http://www.whocc.no/atc_ddd_index/?code=L03AX13&showdescription=yes) | La Mantia L et al. Glatiramer acetate for multiple sclerosis. Cochrane Database Syst Rev 2010;12;5: CD004678 |
| [mycophenolic acid](http://www.whocc.no/atc_ddd_index/?code=L04AA06&showdescription=yes) | Penninga L et al. Calcineurin inhibitor minimisation versus continuation of calcineurin inhibitor treatment for liver transplant recipients. Cochrane Database Syst Rev 2012;14;3: CD008852 |
| [sirolimus](http://www.whocc.no/atc_ddd_index/?code=L04AA10&showdescription=yes) | Webster AC et al. Target of rapamycin inhibitors (TOR-I; sirolimus and everolimus) for primary immunosuppression in kidney transplant recipients. Cochrane Database Syst Rev 2009;21;1: CD004290 |
| [everolimus](http://www.whocc.no/atc_ddd_index/?code=L04AA18&showdescription=yes) | Pascual J et al. Steroid avoidance or withdrawal for kidney transplant recipients. Cochrane Database Syst Rev 2009;21;1: CD005632 |
| [natalizumab](http://www.whocc.no/atc_ddd_index/?code=L04AA23&showdescription=yes) | Pucci E et al. Natalizumab for relapsing remitting multiple sclerosis. Cochrane Database Syst Rev 2011;5;8: CD007621 |
| [etanercept](http://www.whocc.no/atc_ddd_index/?code=L04AB01&showdescription=yes) | Lethaby A et al. Etanercept for the treatment of rheumatoid arthritis. Cochrane Database Syst Rev 2013;31;5: CD004525 |
| [infliximab](http://www.whocc.no/atc_ddd_index/?code=L04AB02&showdescription=yes) | Blumenauer B et al. Infliximab for the treatment of rheumatoid arthritis. Cochrane Database Syst Rev 2009;21;1: CD003785  Behm BW et al. Tumor necrosis factor-alpha antibody for maintenance of remission in Crohn's disease. Cochrane Database Syst Rev 2009;19;4: CD006893 |
| [adalimumab](http://www.whocc.no/atc_ddd_index/?code=L04AB04&showdescription=yes) | Navarro-Sarabia F et al. Adalimumab for treating rheumatoid arthritis. Cochrane Database Syst Rev 2009;21;1: CD005113 |
| [golimumab](http://www.whocc.no/atc_ddd_index/?code=L04AB06&showdescription=yes) | Singh JA et al. Golimumab for rheumatoid arthritis. Cochrane Database Syst Rev 2009;10;11: CD008341 |
| [basiliximab](http://www.whocc.no/atc_ddd_index/?code=L04AC02&showdescription=yes) | Webster AC et al. Interleukin 2 receptor antagonists for kidney transplant recipients. Cochrane Database Syst Rev 2010;17;3: CD003897 |
| [tocilizumab](http://www.whocc.no/atc_ddd_index/?code=L04AC07&showdescription=yes) | Singh JA et al. Tocilizumab for rheumatoid arthritis. Cochrane Database Syst Rev 2010;7;7: CD008331 |
| [tacrolimus](http://www.whocc.no/atc_ddd_index/?code=L04AD02&showdescription=yes) | Haddad E et al. Cyclosporin versus tacrolimus for liver transplanted patients. Cochrane Database Syst Rev 2009;21;1: CD005161  Webster AC et al. Tacrolimus versus cyclosporin as primary immunosuppression for kidney transplant recipients. Cochrane Database Syst Rev 2009;21;1: CD003961 |
| **Systemic infections:** |  |
| zanamivir | Jefferson T et al. Neuraminidase inhibitors for preventing and treating influenza in healthy adults and children. Cochrane Database Syst Rev 2012;18;1: CD008965 |
| abakavir+lamivudin | Shey MS et al. Co-formulated abacavir-lamivudine-zidovudine for initial treatment of HIV infection and AIDS. Cochrane Database Syst Rev 2013;28;3: CD005481 |
| tenofovir+emtricitabin | Okwundu C et al. Antiretroviral pre-exposure prophylaxis (PrEP) for preventing HIV in high-risk individuals. Cochrane Database Syst Rev 2012;11;7: CD007189 |
| **Blood:** |  |
| [antithrombin III](http://www.whocc.no/atc_ddd_index/?code=B01AB02&showdescription=yes) | Afshari A et al. Antithrombin III for critically ill patients. Cochrane Database Syst Rev 2008;16;87: CD005370 |
| [enoxaparin](http://www.whocc.no/atc_ddd_index/?code=B01AB05&showdescription=yes) | Sandercock PAG et al. Low-molecular-weight heparins or heparinoids versus standard unfractionated heparin for acute ischaemic stroke. Cochrane Database Syst Rev 2008;8;8: CD000119 |
| [nadroparin](http://www.whocc.no/atc_ddd_index/?code=B01AB06&showdescription=yes) | Sandercock PAG et al. Anticoagulants for acute ischaemic stroke. Cochrane Database Syst Rev 2009;15;4: CD000024 |
| [fondaparinux](http://www.whocc.no/atc_ddd_index/?code=B01AX05&showdescription=yes) | Sandercock PAG et al. Heparine Low-molecular-weight heparins or heparinoids versus standard unfractionated heparin for acute ischaemic stroke. Cochrane Database Syst Rev 2008;8;8: CD000119 |
| [aprotinin](http://www.whocc.no/atc_ddd_index/?code=B02AB01&showdescription=yes) | Henry DA et al. Anti-fibrinolytic use for minimising perioperative allogeneic blood transfusion. Cochrane Database Syst Rev 2011;16;3: CD001886 |
| [erythropoietin](http://www.whocc.no/atc_ddd_index/?code=B03XA01&showdescription=yes) | Cody JD et al. Frequency of administration of recombinant human erythropoietin for anaemia of end-stage renal disease in dialysis patients. Cochrane Database Syst Rev 2009;21;1: CD003895 |
| [darbepoetin alfa](http://www.whocc.no/atc_ddd_index/?code=B03XA02&showdescription=yes) | Strippoli GFM et al. Haemoglobin and haematocrit targets for the anaemia of chronic kidney disease. Cochrane Database Syst Rev 2010;17;2: CD003967 |
| [amino acids](http://www.whocc.no/atc_ddd_index/?code=B05BA01&showdescription=yes) | Wagner JVE et al. Glutamine supplementation for young infants with severe gastrointestinal disease. Cochrane Database Syst Rev 2012;11;1: CD005947 |
| **Respiratory:** |  |
| [formoterol and other drugs for obstructive airway diseases](http://www.whocc.no/atc_ddd_index/?code=R03AK07&showdescription=yes) | Nannini LJ et al. Combined corticosteroid and long-acting beta-agonist in one inhaler versus inhaled steroids for chronic obstructive pulmonary disease. Cochrane Database Syst Rev 2010;20;1: CD006826 |
| [salmeterol and other drugs for obstructive airway diseases](http://www.whocc.no/atc_ddd_index/?code=R03AK06&showdescription=yes) | Nannini LJ et al. Combined corticosteroid and long-acting beta-agonist in one inhaler versus inhaled steroids for chronic obstructive pulmonary disease. Cochrane Database Syst Rev 2010;20;1: CD006826 |
| [montelukast](http://www.whocc.no/atc_ddd_index/?code=R03DC03&showdescription=yes) | Ducharme FM et al. Addition to inhaled corticosteroids of long-acting beta2-agonists versus anti-leukotrienes for chronic asthma. Cochrane Database Syst Rev 2011;11;5: CD003137 |
| [dornase alfa (desoxyribonuclease)](http://www.whocc.no/atc_ddd_index/?code=R05CB13&showdescription=yes) | Jones AP et al. Dornase alfa for cystic fibrosis. Cochrane Database Syst Rev 2010;17;3: CD001127 |
| **Musculoskeletal:** |  |
| [botulinum toxin](http://www.whocc.no/atc_ddd_index/?code=M03AX01&showdescription=yes) | Costa J et al. Botulinum toxin type A therapy for blepharospasm. Cochrane Database Syst Rev 2009;21;1: CD004900 |
| [clodronic acid](http://www.whocc.no/atc_ddd_index/?code=M05BA02&showdescription=yes) | Wong RKS et al. Bisphosphonates for the relief of pain secondary to bone metastases. Cochrane Database Syst Rev 2009;7;8: CD002068 |
| **Urogenital:** |  |
| [atosiban](http://www.whocc.no/atc_ddd_index/?code=G02CX01&showdescription=yes) | Papatsonis D et al. Oxytocin receptor antagonists for inhibiting preterm labour. Cochrane Database Syst Rev 2010;20;1: CD004452 |
| [cyproterone and estrogen](http://www.whocc.no/atc_ddd_index/?code=G03HB01&showdescription=yes) | Van der Spuy ZM et al. Cyproterone acetate for hirsutism. Cochrane Database Syst Rev 2009;8;7: CD001125 |
| [darifenacin](http://www.whocc.no/atc_ddd_index/?code=G04BD10&showdescription=yes) | Madhuvrata P et al. Which anticholinergic drug for overactive bladder symptoms in adults. Cochrane Database Syst Rev 2012;18;1: CD005429 |
| [finasteride](http://www.whocc.no/atc_ddd_index/?code=G04CB01&showdescription=yes) | Tacklind J et al. Finasteride for benign prostatic hyperplasia. Cochrane Database Syst Rev 2010;6;8: CD006015 |
| [human menopausal gonadotrophin](http://www.whocc.no/atc_ddd_index/?code=G03GA02&showdescription=yes) | Westergaard LW et al. Human menopausal gonadotropin versus recombinant follicle stimulation hormone for ovarian stimulation in assisted reproductive cycles. Cochrane Database Syst Rev2011;16;2 CD003973 |
| [propiverine](http://www.whocc.no/atc_ddd_index/?code=G04BD06&showdescription=yes) | Nabi G et al. Anticholinergic drugs versus placebo for overactive bladder syndrome in adults. Cochrane Database Syst Rev 2009;21;1: CD003781 |
| [solifenacin](http://www.whocc.no/atc_ddd_index/?code=G04BD08&showdescription=yes) | Madhuvrata P et al. Which anticholinergic drug for overactive bladder symptoms in adults. Cochrane Database Syst Rev 2012;18;1: CD005429 |
| [sildenafil](http://www.whocc.no/atc_ddd_index/?code=G04BE03&showdescription=yes) | Kanthapillai P et al. Phosphodiesterase five inhibitors for pulmonary hypertension. Cochrane Database Syst Rev 2009;8;7: CD003562 |
| [tamsulosin](http://www.whocc.no/atc_ddd_index/?code=G04CA02&showdescription=yes) | Wilt TJ et al. Tamsulosin for benign prostatic hyperplasia. Cochrane Database Syst Rev 2011;8:9 |
